# A new computational strategy for identifying essential proteins based on network topological properties and biological information

**DOI:** 10.1371/journal.pone.0182031

**Published:** 2017-07-28

**Authors:** Chao Qin, Yongqi Sun, Yadong Dong

**Affiliations:** Beijing Key Lab of Traffic Data Analysis and Mining, School of Computer and Information Technology, Beijing Jiaotong University, Beijing, China; King’s College London, UNITED KINGDOM

## Abstract

Essential proteins are the proteins that are indispensable to the survival and development of an organism. Deleting a single essential protein will cause lethality or infertility. Identifying and analysing essential proteins are key to understanding the molecular mechanisms of living cells. There are two types of methods for predicting essential proteins: experimental methods, which require considerable time and resources, and computational methods, which overcome the shortcomings of experimental methods. However, the prediction accuracy of computational methods for essential proteins requires further improvement. In this paper, we propose a new computational strategy named CoTB for identifying essential proteins based on a combination of topological properties, subcellular localization information and orthologous protein information. First, we introduce several topological properties of the protein-protein interaction (PPI) network. Second, we propose new methods for measuring orthologous information and subcellular localization and a new computational strategy that uses a random forest prediction model to obtain a probability score for the proteins being essential. Finally, we conduct experiments on four different *Saccharomyces* *cerevisiae* datasets. The experimental results demonstrate that our strategy for identifying essential proteins outperforms traditional computational methods and the most recently developed method, SON. In particular, our strategy improves the prediction accuracy to 89, 78, 79, and 85 percent on the YDIP, YMIPS, YMBD and YHQ datasets at the top 100 level, respectively.

## Introduction

Essential proteins are the proteins that are indispensable to the survival of an organism; therefore, these proteins are considered to be the basis of life. Deleting any of these proteins will lead to cell death [1]. Thus, identifying essential proteins is of great significance, and it will help us understand the minimum requirements for cell life and find novel treatments for diseases [2–4].

To date, many traditional biological methods have been proposed for identifying essential proteins, such as gene knockouts [5], conditional knockouts [6] and RNA interference [7]. These traditional biological methods are time consuming, expensive and not always practical. To overcome the shortcomings of these biological methods, a number of computational methods that only consider the topological properties have been proposed, such as degree centrality (DC) [8], betweenness centrality (BC) [9], eigenvector centrality (EC) [10], subgraph centrality (SC) [11], local average connectivity-based method (LAC) [12], and network centrality (NC) [13]. To further improve the prediction accuracy, Li [14] proposed a method called TP that uses topology potential to identify essential proteins. Although these methods facilitate the detection of essential proteins, they only consider the topological properties of the network, and they do not take the intrinsic properties of individual proteins into account. Consequently, many computational methods combined with biological information have been proposed. Such biological information includes protein complex information, gene expression data, subcellular localization information, orthologous protein information, and so on.

A protein complex is a group of proteins that interact with each other and function as a unit at a given time and place in a certain biological process. It has been proven that essential proteins are more likely to gather in protein complexes [15]. Based on this idea, Li [15] proposed a method called united complex centrality (UC) that integrates protein complex information. Luo [16] proposed a method named LIDC combined with the in-degree centrality of complex (IDC), which measures the in-degree value of a protein in the protein complex. Qin [17] proposed a method named LBCC that integrates the local topological features, global topological features, and protein complex information, where the local topological features are Den_1_ and Den_2_, representing the local densities of networks, and it improved the prediction precision to 75 percent on the YMIPS dataset at the top 100 level.

Gene expression is the process of transcribing and translating genetic information stored in a DNA sequence into functional gene products, which are often proteins, and it is also an important feature for predicting essential proteins. Li [18] proposed a method named PeC that integrates gene expression data for predicting essential proteins. Tang [19] proposed a weighted degree centrality using gene expression data to achieve the reliable prediction of essential proteins. Zhao [20] proposed a method named PEMC that integrates network topology with gene expression profile and protein domain information to construct weighted protein networks for discovering essential proteins.

Some researchers have reported that the locations of proteins are correlated with their essentiality and that essential proteins appear more frequently at specific locations. Zhong [21] proposed a feature selection method for predicting essential proteins, and the results indicated that the subcellular localization information can help increase the prediction accuracy for predicting essential proteins.

Orthologous protein information is another important aspect for identifying essential proteins. Orthologous proteins are proteins that are derived from a common ancestor and generally retain the same or very similar functions. It has been proven that orthologous properties are positively correlated with protein essentiality [22]. Li [23] proposed a method named SON that integrates subcellular localization and orthologous score (OS) information, and this method improved the accuracy of predicting essential proteins to approximately 81 percent on the YDIP dataset at the top 100 level. Li [24] proposed a method named GOS that integrates gene expression, orthology, and subcellular localization information to identify essential proteins.

In this paper, we first introduce several traditional topological properties of protein-protein interaction (PPI) networks, including Laplacian centrality (LC) [25], which is an intermediate measure between global and local properties. We then propose new measures of orthologous score (DOS) and subcellular localization score (SLS), as well as our new prediction method named CoTB, which combines Den_1_, Den_2_, BC, IDC, LC, DOS and SLS and uses a random forest model to obtain a probability score for the proteins being essential.

We conducted our experiments on four different PPI networks of *Saccharomyces* *cerevisiae*, namely, YDIP, YMIPS, YMBD, and YHQ, which will be described in the Experimental data section. The experimental results showed that our method, CoTB, obtained superior performance compared to the traditional measures, including DC, BC, SC, EC, NC, and LAC. CoTB exhibited the best performance and obtained prediction precisions of 89, 78, 79, and 85 percent on the YDIP, YMIPS, YMBD and YHQ datasets at the top 100 level, respectively. In particular, compared to the most recently developed method, SON [23], CoTB improved the prediction precisions by at least 9, 10, 8, 8, 7, and 8 percent on the YDIP dataset at the top 100 to top 600 levels, respectively. Compared to GOS [24], CoTB improved the prediction precisions by at least 6, 6, 9, and 10 percent on the YDIP dataset at the top 300 to top 600 levels, respectively. Compared to our LBCC [17], CoTB improved the prediction precisions by at least 20, 4, 21, and 500 percent on the YDIP, YMIPS, YMBD and YHQ datasets at the top 100 level, respectively.

## Preliminaries

A PPI network is represented as an undirected simple graph *G*(*V*, *E*) with a set of nodes (proteins) *V* and a set of edges *E* (interactions). Let *N*_*v*_ denote the set of neighbour nodes of node *v*, |*N*_*v*_| denote the number of neighbours of node *v* and *G*[*S*] denote the induced subgraph of *G* on node set *S*. The definitions of several topological properties of a PPI network are as follows.

*Degree centrality* (*DC*). The DC of a node *v* is denoted as the total number of its neighbour nodes, and it is denoted as

$$\begin{array}{c} DC(v)=deg(v), \end{array}$$

where *deg*(*v*) is the number of its incident edges.

*Betweenness centrality* (*BC*). The BC of a node *v* is calculated based on the shortest paths, and it is denoted as

$$\begin{array}{c} BC\left(v\right)=\sum_{s}\sum_{t}\frac{\sigma_{st}\left(v\right)}{\sigma_{st}},s\neq t\neq v\in V, \end{array}$$

where *σ*_*st*_ is the total number of shortest paths from *s* to *t* and *σ*_*st*_(*v*) is the total number of shortest paths passing through *v* from *s* to *t*.

*Eigenvector centrality* (*EC*). The EC of a node *v* is calculated based on the adjacency matrix of the network, and it is denoted as

$$\begin{array}{c} EC\left(v\right)=\alpha_{max}\left(v\right), \end{array}$$

where *α*_*max*_ is the eigenvector corresponding to the largest eigenvalue of the adjacency matrix and *α*_*max*_(*v*) is the *v*th component of *α*_*max*_.

*Local average connectivity centrality* (*LAC*). The LAC of a node *v* describes the closeness of its neighbours, and it is denoted as

$$\begin{array}{c} LAC\left(v\right)=\frac{\sum_{u\in N_{v}}deg^{C_{v}}\left(u\right)}{|N_{v}|},u\in N_{v}, \end{array}$$

where *C*_*v*_ is the induced subgraph of *G* on node set *N*_*v*_ and $deg^{C_{v}}\left(u\right)$ is the number of its neighbour nodes in *C*_*v*_.

*Neighbourhood centrality* (*NC*). The NC of a node *v* considers the importance of the relationship between *v* and its neighbours, and it is denoted as

$$\begin{array}{c} NC\left(v\right)=\sum_{u\in N_{v}}\frac{z_{v,u}}{min(d_{v}-1,d_{u}-1)}, \end{array}$$

where *z*_*v*,*u*_ is the number of common neighbour vertices of *v* and *u* and *d*_*v*_ and *d*_*u*_ are the degrees of nodes *v* and *u*, respectively.

*Subgraph centrality* (*SC*). The SC of a node *v* measures the participation of a node in all subgraphs of the network, and it is denoted as

$$\begin{array}{c} SC\left(v\right)=\sum_{k=0}^{\infty }\frac{\mu_{k}\left(v\right)}{k!}, \end{array}$$

where *μ*_*k*_(*v*) is the number of circles starting and ending at *v* with length *k*.

*Laplacian centrality* (*LC*). For a graph *G* with *n* nodes, let *W*(*G*) be the adjacency matrix of size *n* by *n*, and let *X*(*G*) be a matrix as follows:

$$\begin{array}{c} \left(\begin{array}{cccc} x_{1} & 0 & \ldots & 0\\ 0 & x_{2} & \ldots & 0\\ . & . & . & .\\ 0 & 0 & \ldots & x_{n} \end{array}\right) \end{array}$$

where *x*_*i*_ is the number of neighbours of node *i*. The LC *LC*(*v*_*i*_, *G*) of vertex *v*_*i*_ is defined as

$$\begin{array}{c} LC\left(v_{i},G\right)=\frac{E_{L}\left(G\right)-E_{L}\left(G_{i}\right)}{E_{L}\left(G\right)}, \end{array}$$

$$\begin{array}{c} E_{L}\left(G\right)=\sum_{i=1}^{n}\lambda_{i}^{2}, \end{array}$$

where *G*_*i*_ is the graph obtained by deleting *v*_*i*_ from G and *λ*_*i*_ is the eigenvalues of the matrix *L*(*G*) = *X*(*G*) − *W*(*G*).

*In*-*degree centrality of complex* (*IDC*). The IDC of a node *v* measures the sum of degrees of node *v* in different protein complexes, and it is denoted as

$$\begin{array}{c} IDC\left(v\right)=\sum_{i\in ComplexSet(v)}IN-Degree{\left(v\right)}_{i}, \end{array}$$

where *ComplexSet*(*v*) is the set of protein complexes with protein *v* and *IN*-*Degree*(*v*)_*i*_ is the value of *DC*(*v*) for the *i*−th protein complex in *ComplexSet*(*v*).

*Den*_1_(*v*). For a node *v*, *Den*_1_(*v*) is the ratio of the number of edges to the number of all possible edges of the induced subgraph in *G* by the node set *N*_*v*_ ∪ {*v*}, and it is denoted as

$$\begin{array}{c} Den_{1}\left(v\right)=\frac{2|E(H)|}{\left|V(H)|(|V(H)|-1\right)}, \end{array}$$

where *H* denotes the induced subgraph of *G*[*N*_*v*_ ∪ {*v*}].

*Den*_2_(*v*). For a node *v*, let *M*_*u*_ be the node set for which the distance to *v* is 2. *Den*_2_(*v*) is the ratio of the number of edges to the number of all possible edges of the induced subgraph in *G* by the node set *M*_*u*_ ∪ *N*_*v*_ ∪ {*v*}, and it is denoted as

$$\begin{array}{c} Den_{2}\left(v\right)=\frac{2|E(H)|}{\left|V(H)|(|V(H)|-1\right)}, \end{array}$$ (12)

where *H* denotes the induced subgraph of *G*[*M*_*u*_ ∪ *N*_*v*_ ∪ {*v*}].

## Methods

### New measure of orthologous score

Orthologous proteins are proteins that are derived from a common ancestor and generally retain the same or very similar functions. It has been proven that orthologous properties are positively correlated with protein essentiality [22]. The greater the number of reference organisms in which such a protein appears, the more essential the protein is.

For a protein *v*, its orthologous score *OS*(*v*) from [22] is the number of reference organisms where it appears, and it is denoted as

$$\begin{array}{c} OS\left(v\right)=\sum_{i=1}^{s}T_{i}\left(v\right), \end{array}$$

$$\begin{array}{c} T_{i}\left(v\right)=\{\begin{array}{cc} 1 & v\in o(i)\\ 0 & v\notin o(i) \end{array}, \end{array}$$

where *s* is the number of reference organisms and *o*(*i*) is the set of nodes (proteins) in the *i*th reference organism.

In this paper, we define a new measure of orthologous score *DOS*(*v*) as

$$\begin{array}{c} DOS(v)=a*DC(v)+OS(v), \end{array}$$

where *a* is a scaling parameter that ranges from 0.1 to 1. Through a large number of experiments for identifying essential proteins on four different testing datasets, we found that our new computational strategy, CoTB, which will be described in the following, obtains the best performance when *a* is set to 0.1.

### New measure of subcellular localization score

The localization of proteins is the location in cells where a protein appears. It has been proven that the localization of proteins is an important factor for determining protein essentiality [21, 23, 26, 27], and statistical results show that essential proteins are more likely to exist in specific cellular locations. For example, many important biological processes, such as DNA replication and mRNA synthesis, usually occur in the nuclear.

In this section, we propose a new measure of subcellular localization score (SLS). For a protein *v*, we define its SLS as the sum of the subcellular localization coefficient (SLC) of each subcellular localization,

$$\begin{array}{c} SLS\left(v\right)=\sum_{v\in s(l)}SLC\left(l\right), \end{array}$$

where *s*(*l*) is the set of proteins in the *l* subcellular location and *SLC*(*l*) is the SLC, which is defined as

$$\begin{array}{c} SLC\left(l\right)=\frac{t_{l}}{t}-\frac{a_{l}}{a}, \end{array}$$

where *a*_*l*_ is the total number of proteins in the *l* subcellular location and *a* is the total number of proteins. The values of *t*_*l*_ and *t* are obtained after we rank the proteins in descending order by the values of a certain network topology attribute. In [23], Li selected the top 5% proteins as the essential proteins, so we selected the top 5% proteins as essential proteins, that is, *t*_*l*_ is the number of proteins in the *l* subcellular location from the top 5% proteins, and *t* is the number of the top 5% proteins. We think that the importance of the *l* subcellular location is directly proportional to the number of proteins from the top 5% in the *l* subcellular location. If *SLC*(*l*) is greater than 0, it means that there are more essential proteins appearing in the *l* subcellular location. If *SLC*(*l*) is less than 0, it means that essential proteins rarely appear in the *l* subcellular location.

Because LBCC is one of the most effective methods for identifying essential proteins, we select LBCC [17] to rank proteins in descending order. *LBCC*(*v*) is defined as

$$\begin{array}{c} LBCC\left(v\right)=\log Den_{1}\left(v\right)+4*\log Den_{2}\left(v\right)+3*\log IDC\left(v\right)+\log BC\left(v\right). \end{array}$$

### New computational strategy: CoTB

In this section, we propose our new computational strategy, CoTB. This strategy combines Den_1_, Den_2_, BC, LC, IDC, SLS and DOS. CoTB is based on the following basic concepts:

1. Den_1_ and Den_2_, which are two types of densities, represent the local properties of a PPI network. *Den*_1_(*v*) measures the density of the induced subgraph on the node set of node *v* and its neighbour nodes. *Den*_2_(*v*) measures the density of the induced subgraph on the node set of node *v* and nodes whose distance to node *v* is less than 3.
2. BC represents a global property of a PPI network. A node with a high BC will have more influence on the transfer of information through the network.
3. LC represents an intermediate attribute between the global and local properties used to measure the importance of a node, and it provides more structural information about the connectivity and density around a node.
4. IDC is another topological property that represents the protein complex information, and it has been proven that essential proteins are more likely to gather in protein complexes.
5. SLS is an intrinsic feature of a protein that represents a correlation between the position of a protein in the cell and the protein being essential.
6. DOS is also an intrinsic feature of a protein, and the larger the value is, the more important it is.

To take advantage of these seven attributes, we use the machine learning method random forest [28], which is an efficient method for investigating classification problems, to obtain the probability scores for predicting essential proteins. This method is implemented using the WEKA software package [29], and the number of generated trees is set to be 1000. We then use three of the four datasets as the training set and the remaining one as the testing set, which will be described in the following section. Finally, the proteins are sorted in descending order according to the values of the probability scores for proteins being essential.

## Results and discussion

### Experimental data

We performed experiments based on *Saccharomyces* *cerevisiae* data because its PPI and biological information data were more reliable and complete compared to those of other species, and it has also been widely used in the study of discovering essential proteins. We selected four different datasets from the DIP database [30], the MIPS database (Mammalian Protein-Protein Interaction Database) [31], and the website of the Mark Gerstein Lab (gersteinlab.org), which were denoted as YDIP (S1 Text), YMIPS (S2 Text), YMBD (S3 Text) and YHQ (S4 Text), respectively. The datasets of essential proteins (S1 Excel) were collected from the databases of DEG (Database of Essential Genes) [32], MIPS [31], SGD (Saccharomyces Genome Database) [33], and SGDP (Saccharomyces Genome Deletion Project) [34]. The datasets of protein complexes (S2 Excel) were collected from CM425 [35], CM270 [31], CYC428 and CYC408 [36, 37].

The YDIP dataset contains a total of 5093 proteins, 24743 edges and 1167 essential proteins. The YMIPS dataset contains 4546 proteins, 12319 edges and 1016 essential proteins. YMBD, which was collected from MIPS, BIND and DIP, includes 2559 proteins, 11835 interactions, and 763 essential proteins. The YHQ dataset was constructed by Yu et al. [38], and it contains 4743 proteins, 23294 interactions and 1108 essential proteins. The detailed information of the YDIP, YMIPS, YMBD and YHQ datasets are presented in Table 1.

**Table 1:** Information of the YDIP, YMIPS, YMBD, and YHQ datasets.

Dataset	Proteins	Interactions	Essential proteins
YDIP	5093	24743	1167
YMIPS	4546	12319	1016
YMBD	2559	11835	763
YHQ	4743	23294	1108

The dataset of orthologous proteins was downloaded from the InParanoid database [39], and it contains 99 reference organisms of *Saccharomyces* *cerevisiae*. The subcellular localization dataset of *Saccharomyces* *cerevisiae* was downloaded from the COMPARTMENTS database [40]. After preprocessing, a total of 4849 different proteins remained, in which there were 1140 essential proteins and 11 different localizations, including cell wall, plasma membrane, cytosol, cytoskeleton, vacuole, peroxisome, Golgi apparatus, endosome, endoplasmic reticulum, nucleus, and mitochondrion.

### Comparison with other prediction measures

In this section, we compare CoTB with several existing methods on the four datasets mentioned in the Experimental data section. The algorithms for LIDC, LBCC and SON were implemented according to [16, 17] and [23], respectively, and the other algorithms were implemented using CytoNCA [41], which is a Cytoscape plugin for centrality analysis of biological networks. We selected three of the four datasets as the training set and the remaining one as the testing set, and we selected six levels from the top 100 to top 600 as candidate essential proteins.

**Fig 1:**
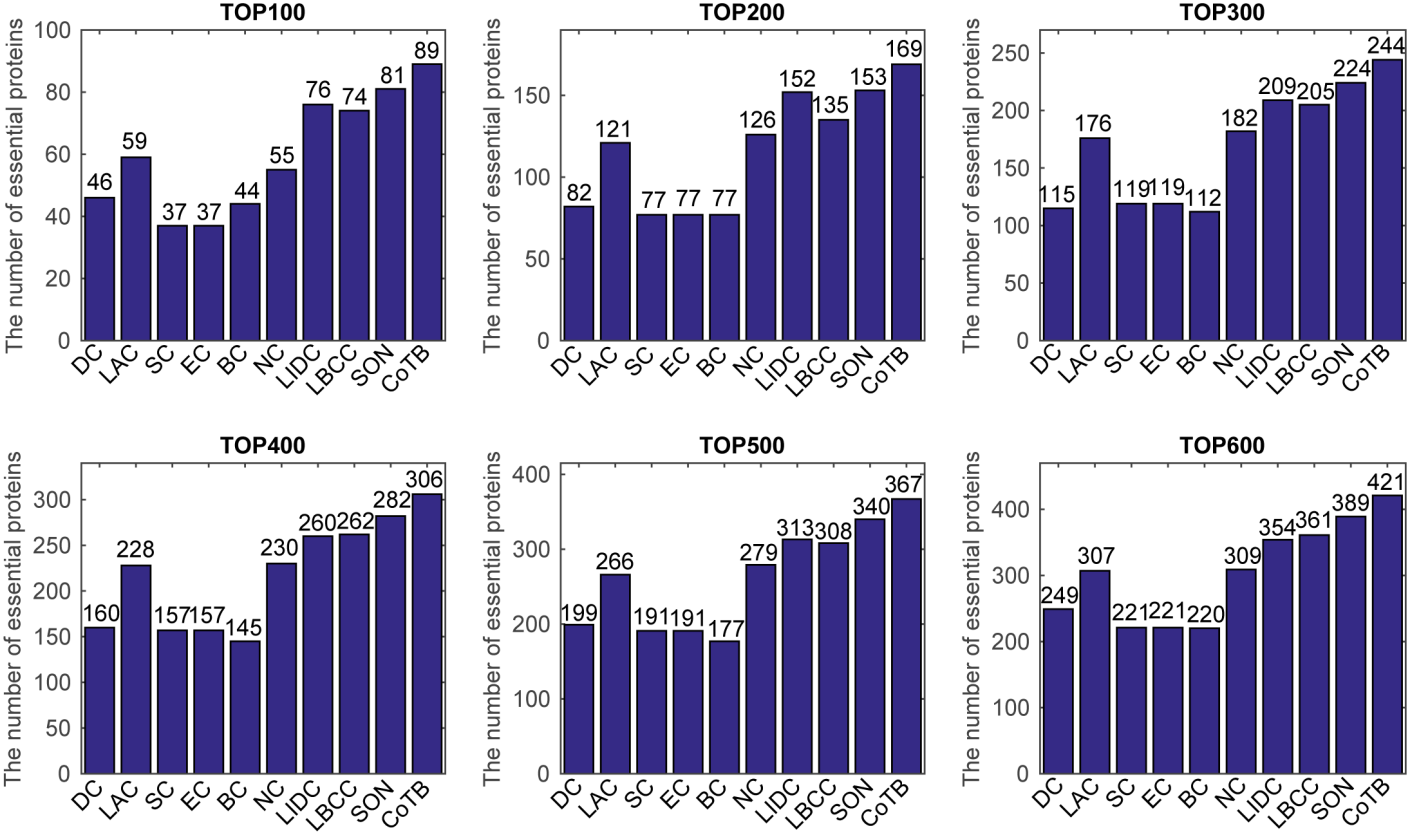
The number of essential proteins predicted by CoTB and the other nine methods at six levels for the YDIP network.

The prediction results are shown in Fig 1 for when YDIP was considered as the testing set and the other three datasets were considered as the training set. CoTB improved the prediction precisions to approximately 89, 85, 82, 77, 74, and 71 percent at six levels. CoTB exhibited superior performance compared with the other methods, and it increased the prediction precisions by more than 20, 25, 19, 16, 19, and 16 percent at six levels compared with LBCC. Moreover, CoTB improved the prediction precisions by more than 9, 10, 8, 8, 7, and 8 percent at six levels compared to the most recently developed method, SON.

**Fig 2:**
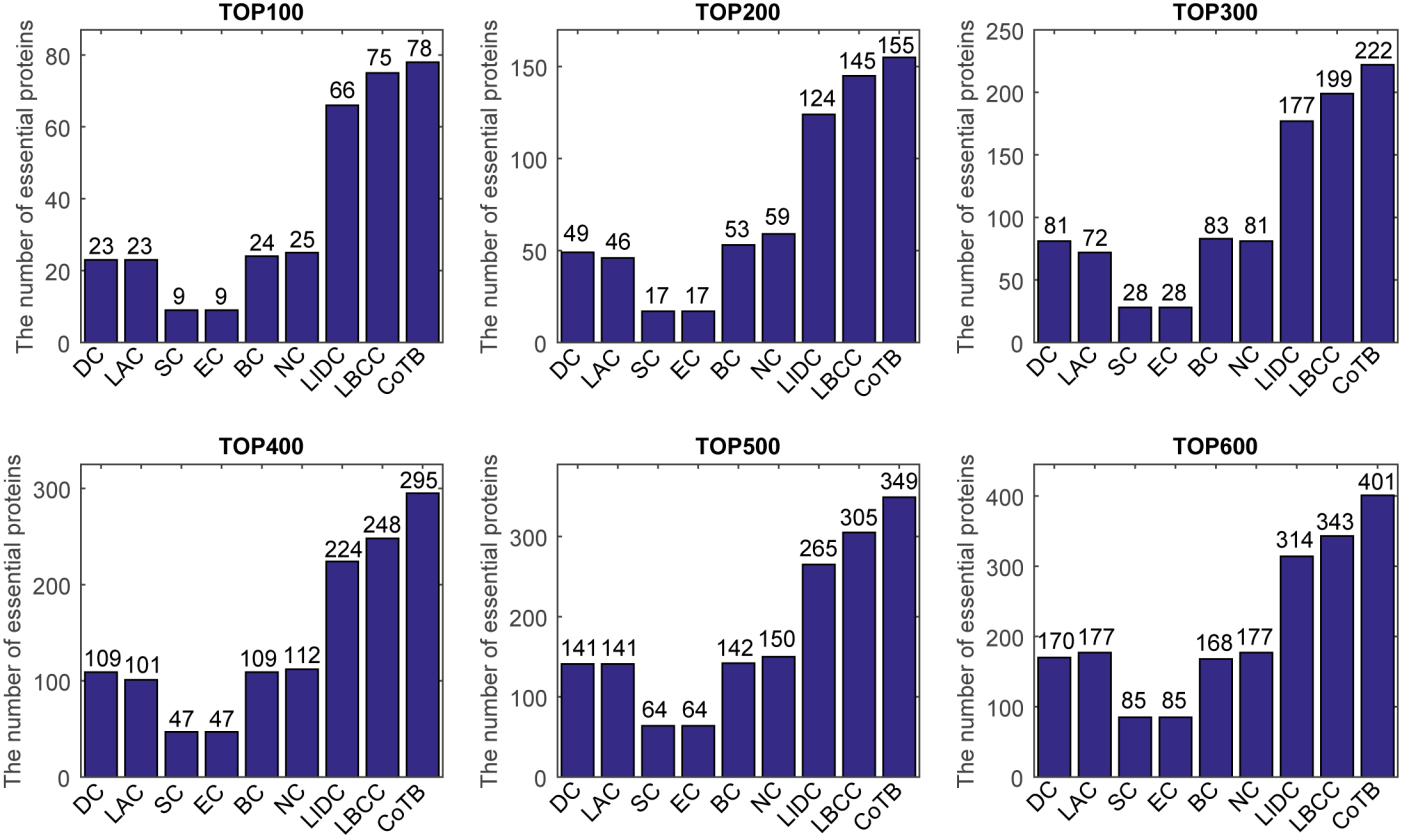
The number of essential proteins predicted by CoTB and the other eight methods at six levels for the YMIPS network.

The prediction results are shown in Fig 2 for when YMIPS was considered as the testing set and the other three datasets were considered as the training set. CoTB improved the prediction precisions to approximately 78, 78, 74, 74, 70, and 67 percent at the six levels. CoTB achieved the best results compared to the other methods, and it increased the prediction precisions by more than 4, 6, 11, 18, 14, and 16 percent at the six levels compared to LBCC, which obtained the best results except for CoTB.

**Fig 3:**
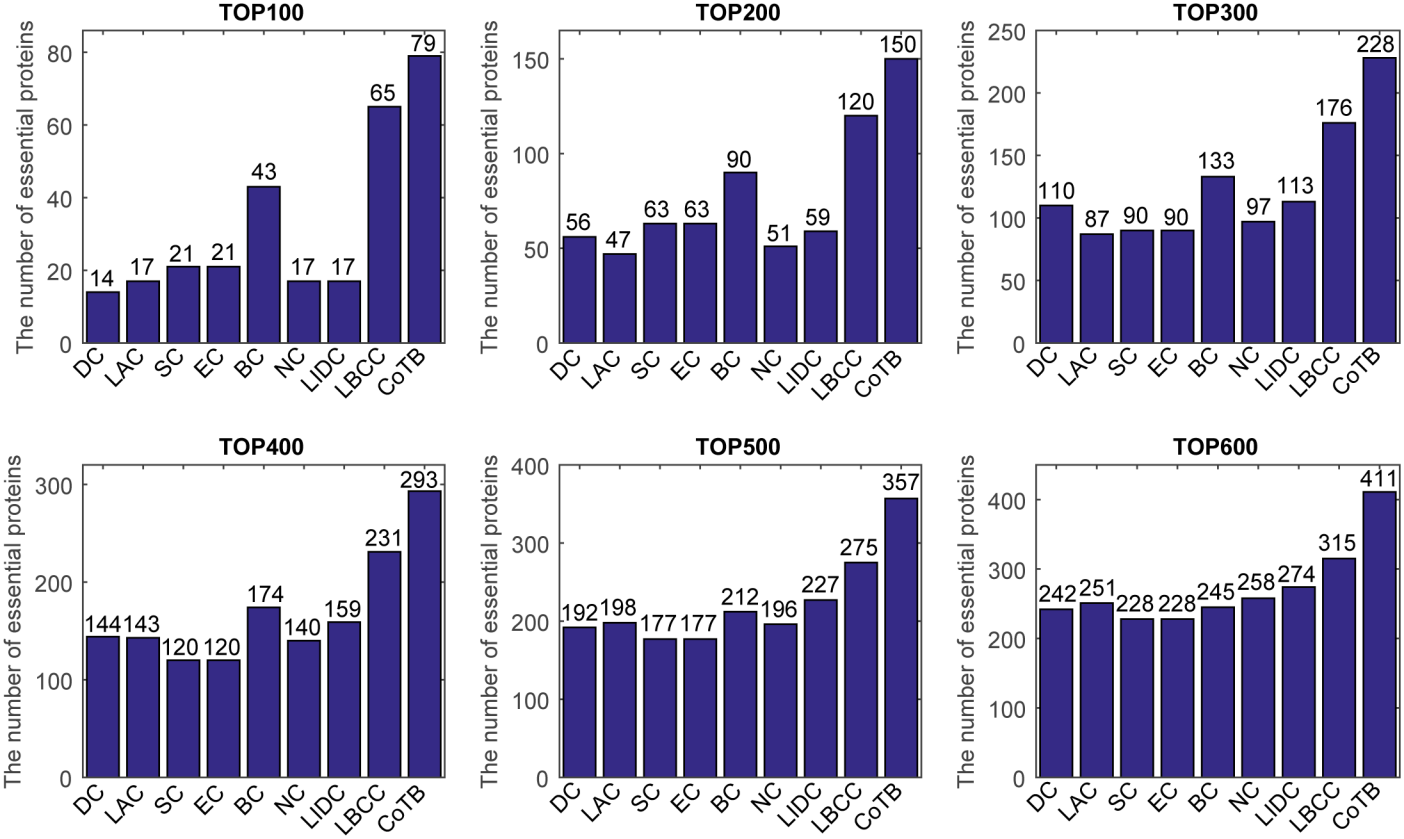
The number of essential proteins predicted by CoTB and the other eight methods at six levels for the YMBD network.

The prediction results are shown in Fig 3 for when YMBD was considered as the testing set and the other three datasets were considered as the training set. CoTB improved the prediction precisions to approximately 79, 75, 76, 74, 72, and 69 percent at six levels from the top 100 to top 600, respectively. CoTB obtained the best results, and it increased the prediction precisions by more than 21, 25, 29, 26, 29, and 30 percent at six levels compared to LBCC.

**Fig 4:**
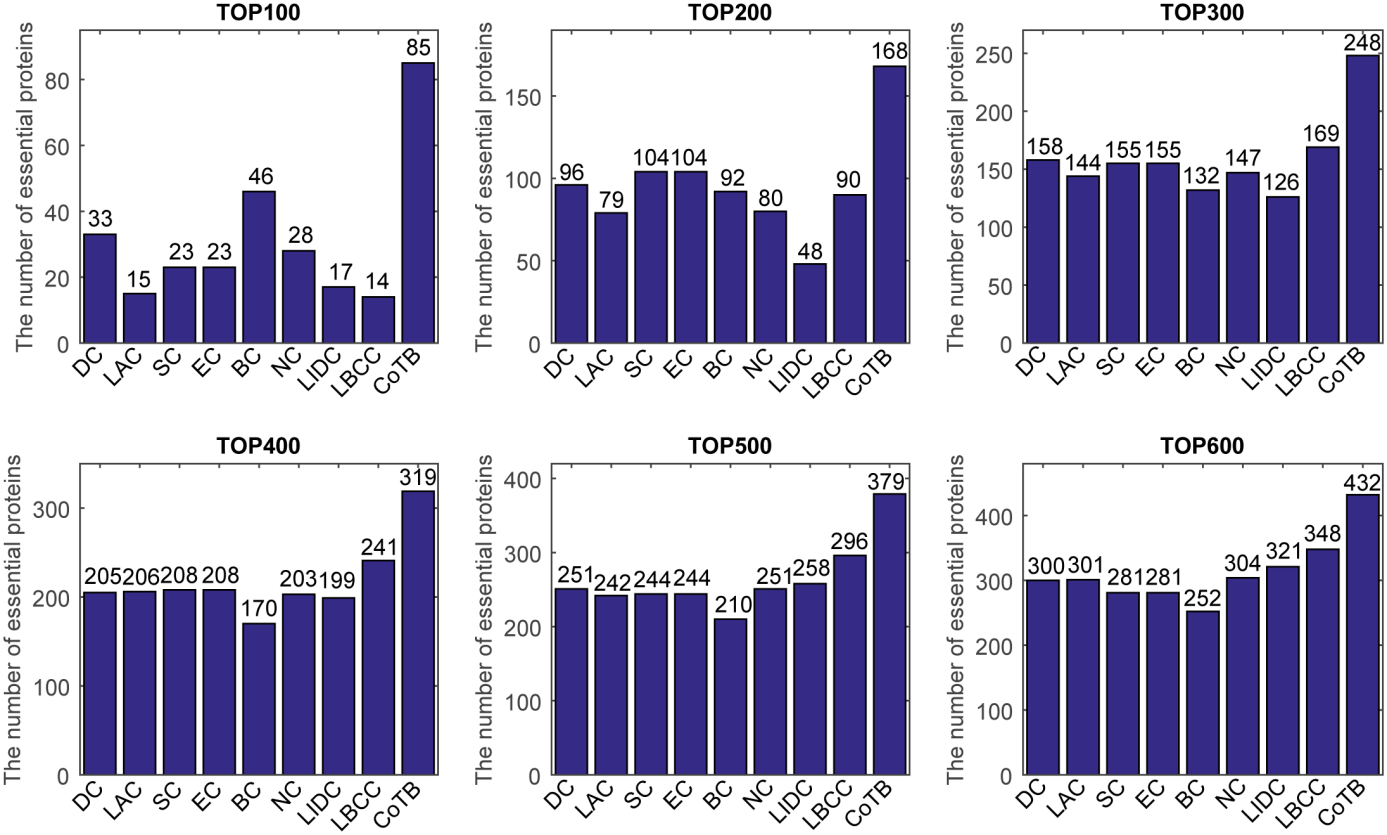
The number of essential proteins predicted by CoTB and the other eight methods at six levels for the YHQ network.

The prediction results are shown in Fig 4 for when YHQ was considered as the testing set and the other three datasets were considered as the training set. CoTB improved the prediction precisions to approximately 85, 84, 83, 80, 76, and 72 percent at six levels. Except for CoTB, the largest numbers of true essential proteins predicted at six levels from the top 100 to top 600 were 46 (BC), 104 (SC, EC), 169 (LBCC), 241 (LBCC), 296 (LBCC), and 348 (LBCC). CoTB increased the prediction precisions by more than 84, 61, 46, 32, 28, and 24 percent compared to the largest numbers at the six levels from the top 100 to top 600, respectively.

### Validation using six statistical measures and precision-recall curves

In this section, we compare CoTB with the other methods using six statistical measures: sensitivity (SN), specificity (SP), positive predictive value (PPV), negative predictive value (NPV), F-measure, and accuracy (ACC) (see references [13, 16]). Let TP be the number of essential proteins predicted as essential proteins, FP be the number of nonessential proteins predicted as essential proteins, TN be the number of nonessential proteins predicted as nonessential proteins, and FN be the number of essential proteins predicted as nonessential proteins. Then, the six statistical measures are defined as follows:

$$\begin{array}{c} SN=\frac{TP}{TP+FN}, \end{array}$$

$$\begin{array}{c} SP=\frac{TN}{TN+FP}, \end{array}$$

$$\begin{array}{c} PPV=\frac{TP}{TP+FP}, \end{array}$$

$$\begin{array}{c} NPV=\frac{TN}{TN+FN}, \end{array}$$

$$\begin{array}{c} F-measure=\frac{2*SN*PPV}{SN+PPV}, \end{array}$$

$$\begin{array}{c} ACC=\frac{TP+TN}{TP+TN+FP+FN}. \end{array}$$

We sorted the proteins in descending order according to the values of the corresponding measures and chose the top 20 percent proteins as essential proteins, and the other proteins were considered to be nonessential proteins. The results are presented in Table 2, which shows that the values of the six statistical measures for CoTB are consistently higher than those of the other methods on four networks, and CoTB improved the values of *SN*, *SP*, *PPV*, *NPV*, *F* − *measure*, and *ACC* by more than 5.4, 0.9, 5.2, 0.9, 5.2, and 1.5 percent compared to SON on the YDIP dataset.

**Table 2:** Comparative analysis of CoTB and the other methods in terms of SN, SP, PPV, NPV, F-measure, and ACC with four different testing datasets.

Dataset	Methods	SN	SP	PPV	NPV	F-measure	ACC
YDIP	DC	0.354	0.846	0.406	0.815	0.378	0.733
	LAC	0.405	0.861	0.465	0.830	0.433	0.757
	SC	0.323	0.837	0.370	0.806	0.345	0.719
	EC	0.323	0.837	0.370	0.806	0.345	0.719
	BC	0.308	0.832	0.354	0.802	0.330	0.712
	NC	0.398	0.859	0.456	0.827	0.425	0.753
	LIDC	0.446	0.873	0.511	0.841	0.476	0.775
	LBCC	0.446	0.873	0.512	0.841	0.477	0.776
	SON	0.497	0.888	0.570	0.856	0.531	0.799
	**CoTB**	**0.524**	**0.896**	**0.600**	**0.864**	**0.559**	**0.811**
YMIPS	DC	0.252	0.815	0.282	0.791	0.266	0.689
	LAC	0.269	0.820	0.300	0.796	0.284	0.697
	SC	0.139	0.782	0.155	0.759	0.146	0.639
	EC	0.139	0.782	0.155	0.759	0.146	0.639
	BC	0.249	0.814	0.278	0.790	0.263	0.688
	NC	0.281	0.824	0.315	0.799	0.297	0.702
	LIDC	0.423	0.864	0.473	0.839	0.447	0.766
	LBCC	0.430	0.866	0.481	0.841	0.454	0.769
	**CoTB**	**0.552**	**0.901**	**0.617**	**0.875**	**0.583**	**0.823**
YMBD	DC	0.260	0.825	0.387	0.724	0.311	0.657
	LAC	0.271	0.830	0.404	0.728	0.325	0.664
	SC	0.239	0.816	0.355	0.716	0.285	0.644
	EC	0.239	0.816	0.355	0.716	0.285	0.644
	BC	0.283	0.835	0.422	0.733	0.339	0.671
	NC	0.266	0.828	0.396	0.726	0.318	0.660
	LIDC	0.308	0.846	0.459	0.742	0.369	0.685
	LBCC	0.372	0.873	0.555	0.766	0.445	0.724
	**CoTB**	**0.480**	**0.919**	**0.715**	**0.806**	**0.574**	**0.788**
YHQ	DC	0.401	0.861	0.468	0.825	0.432	0.754
	LAC	0.431	0.870	0.504	0.834	0.465	0.768
	SC	0.326	0.838	0.380	0.803	0.351	0.719
	EC	0.326	0.838	0.380	0.803	0.351	0.719
	BC	0.330	0.840	0.386	0.804	0.356	0.721
	NC	0.426	0.869	0.497	0.832	0.459	0.765
	LIDC	0.449	0.876	0.524	0.839	0.483	0.776
	LBCC	0.449	0.876	0.524	0.839	0.483	0.776
	**CoTB**	**0.520**	**0.897**	**0.607**	**0.860**	**0.560**	**0.809**

**Fig 5:**
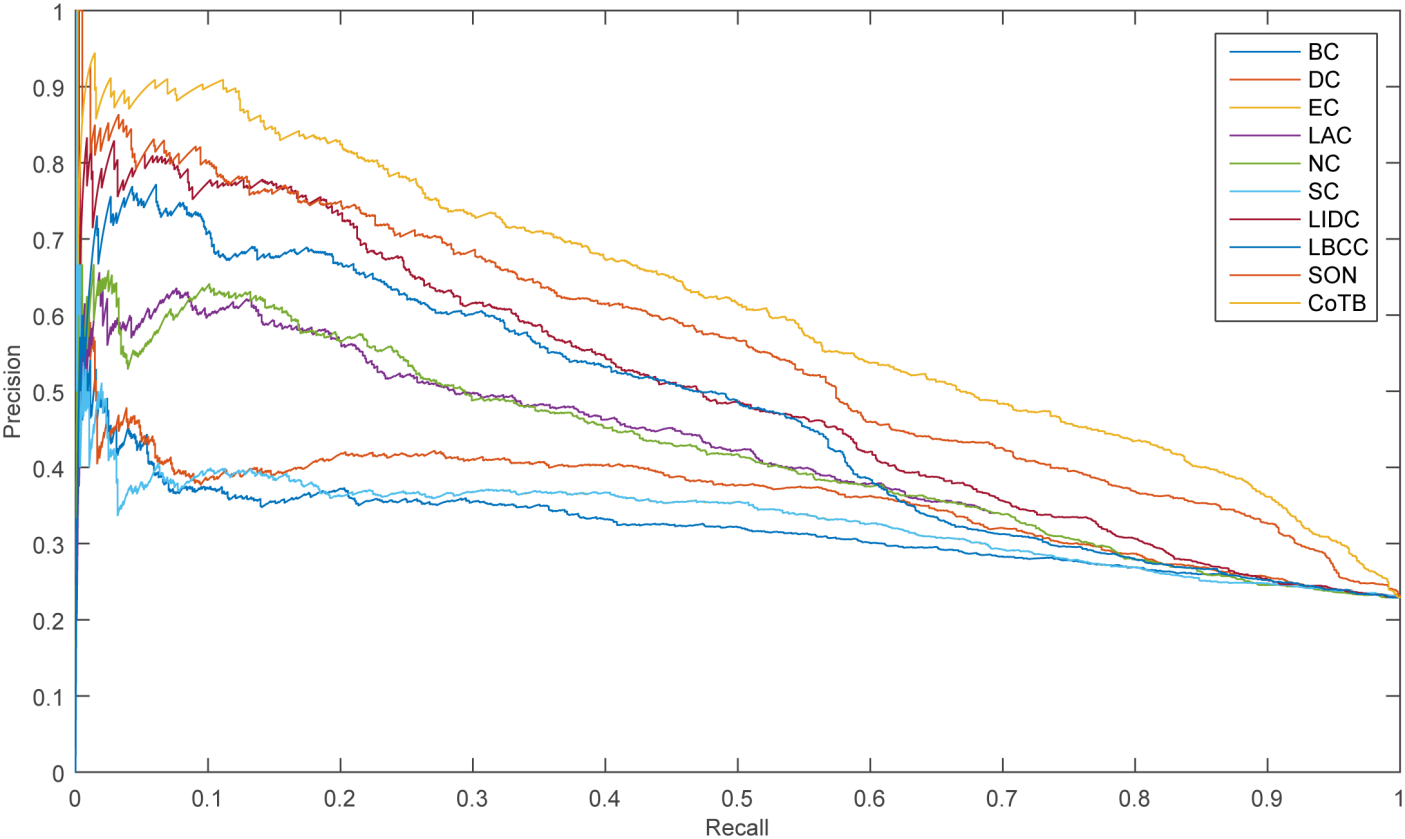
PR curves of CoTB and the other methods for the YDIP network.

**Fig 6:**
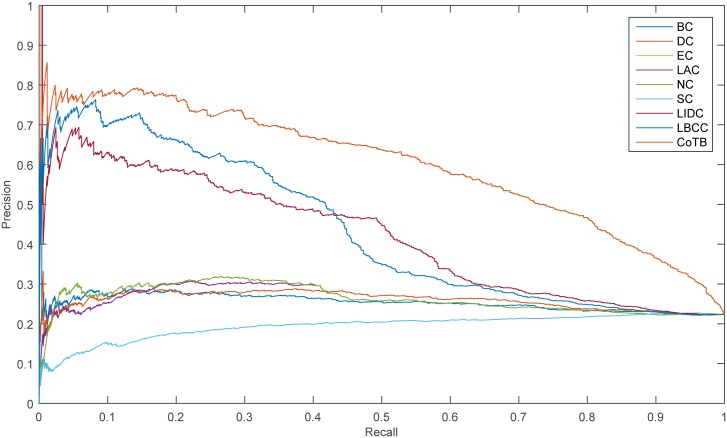
PR curves of CoTB and the other methods for the YMIPS network.

**Fig 7:**
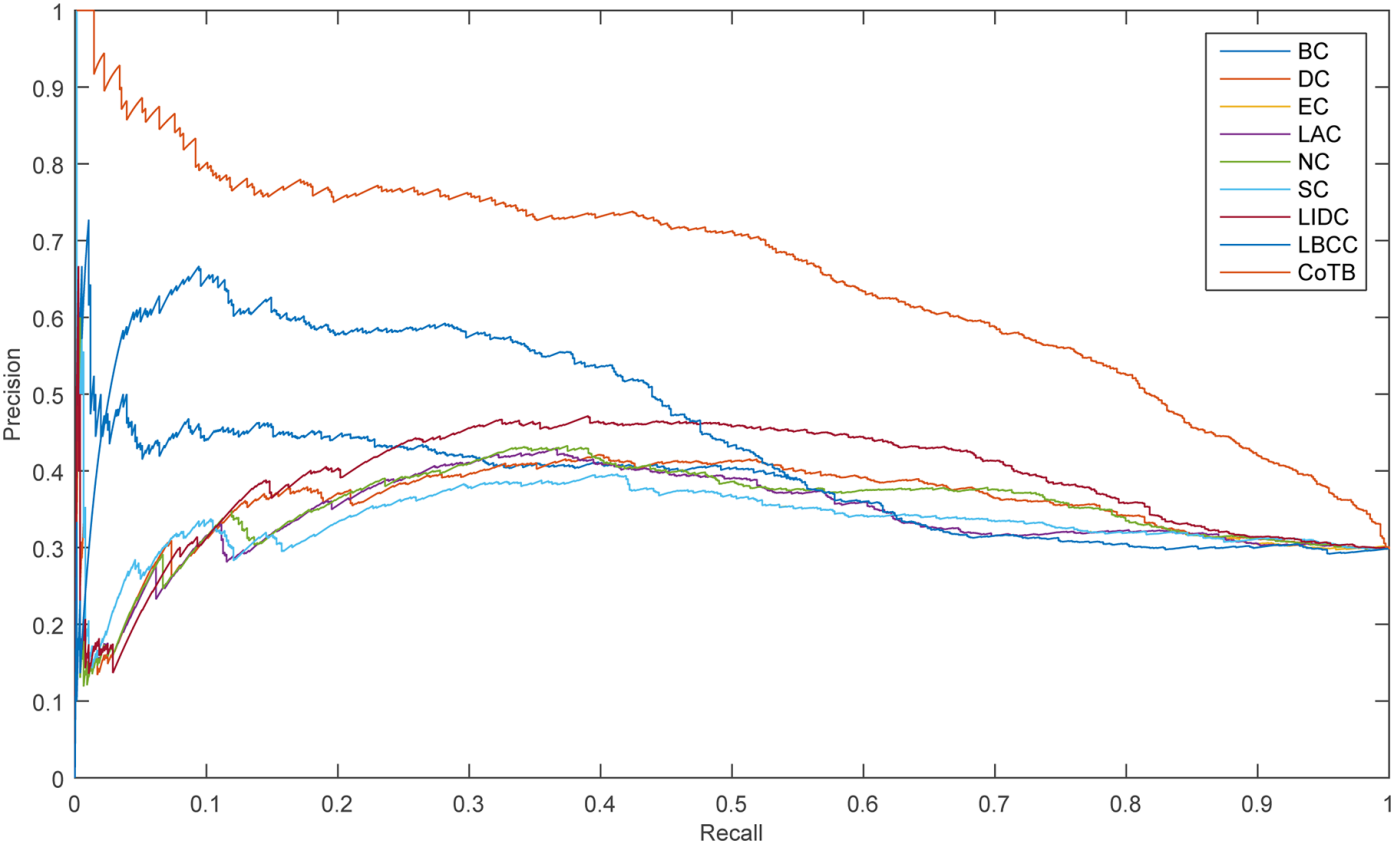
PR curves of CoTB and the other methods for the YMBD network.

**Fig 8:**
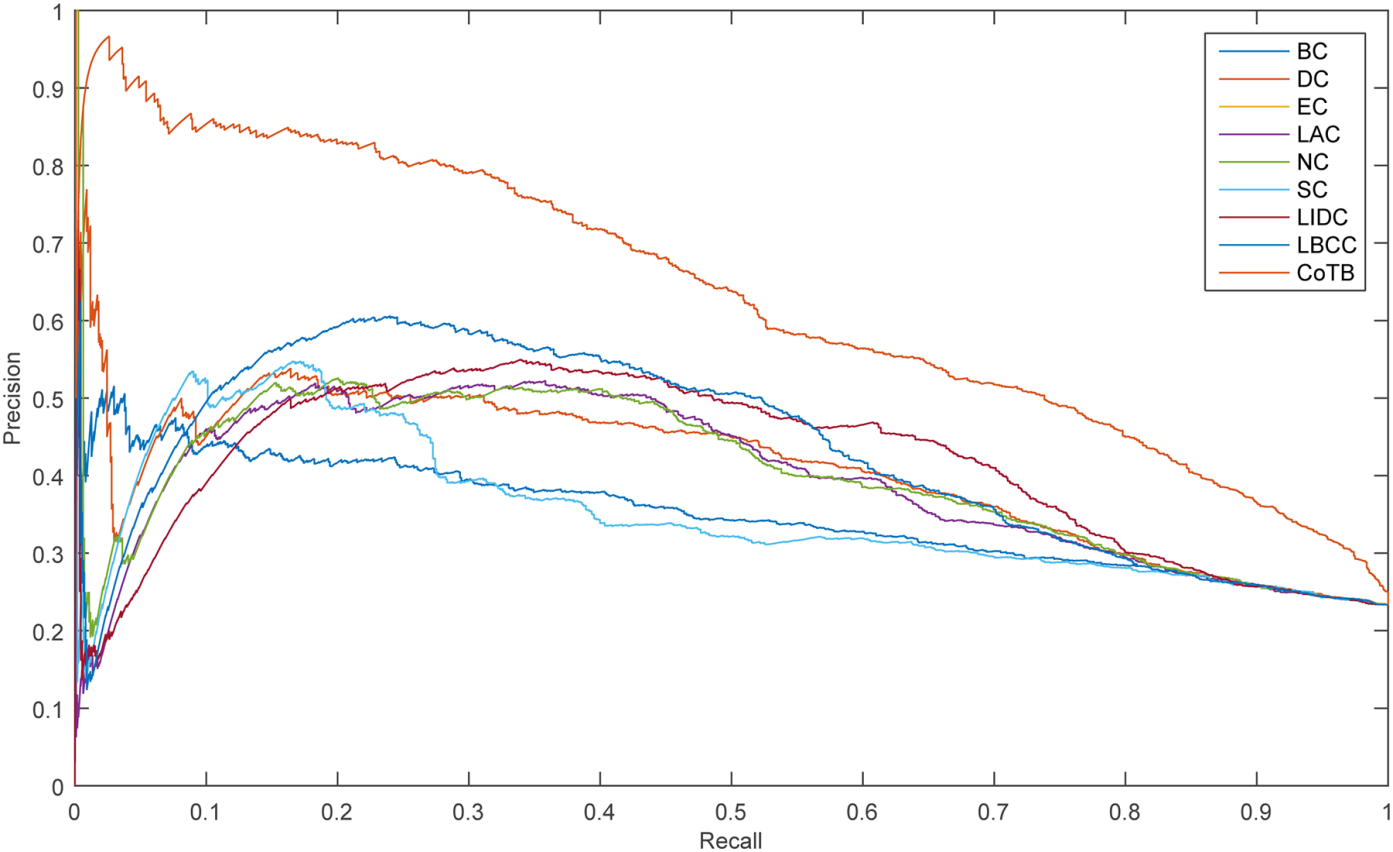
PR curves of CoTB and the other methods for the YHQ network.

The precision-recall curve is used for assessing the stability of the methods, and it is obtained by plotting

$$\begin{array}{c} Precision\left(n\right)=\frac{TP(n)}{TP(n)+FP(n)}, \end{array}$$

$$\begin{array}{c} Recall\left(n\right)=\frac{TP(n)}{P}, \end{array}$$

where *TP*(*n*) is the number of true essential proteins identified correctly and *FP*(*n*) is the number of true essential proteins identified incorrectly among the top n proteins, and *P* is the number of true essential proteins in total. The results are shown in Figs 5–8. As shown, CoTB performed significantly better than SON, LBCC, and the other methods.

### Validation using jackknife methodology

**Fig 9:**
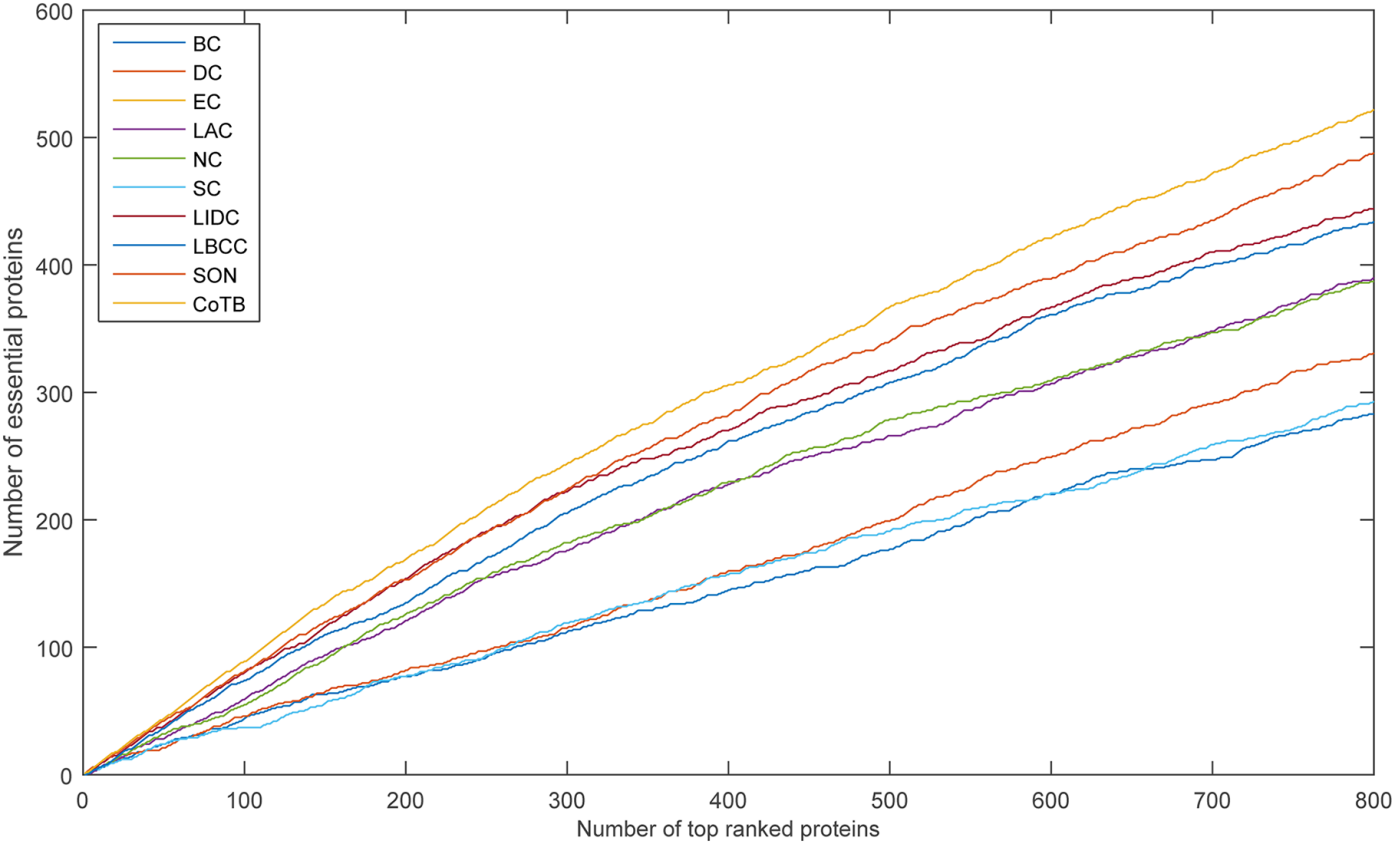
Jackknife curves of CoTB and the other methods for the YDIP network.

**Fig 10:**
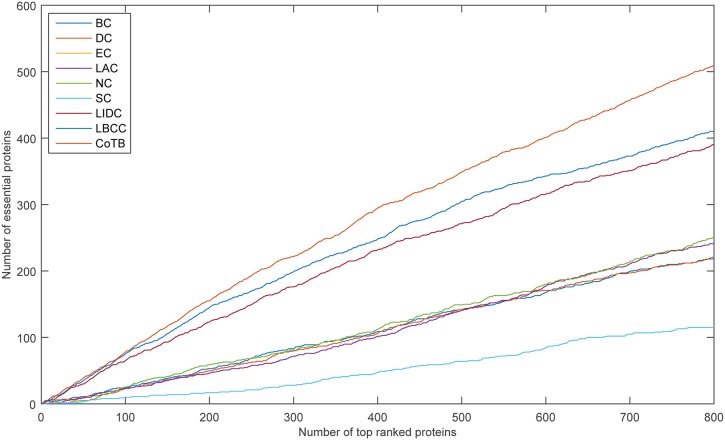
Jackknife curves of CoTB and the other methods for the YMIPS network.

**Fig 11:**
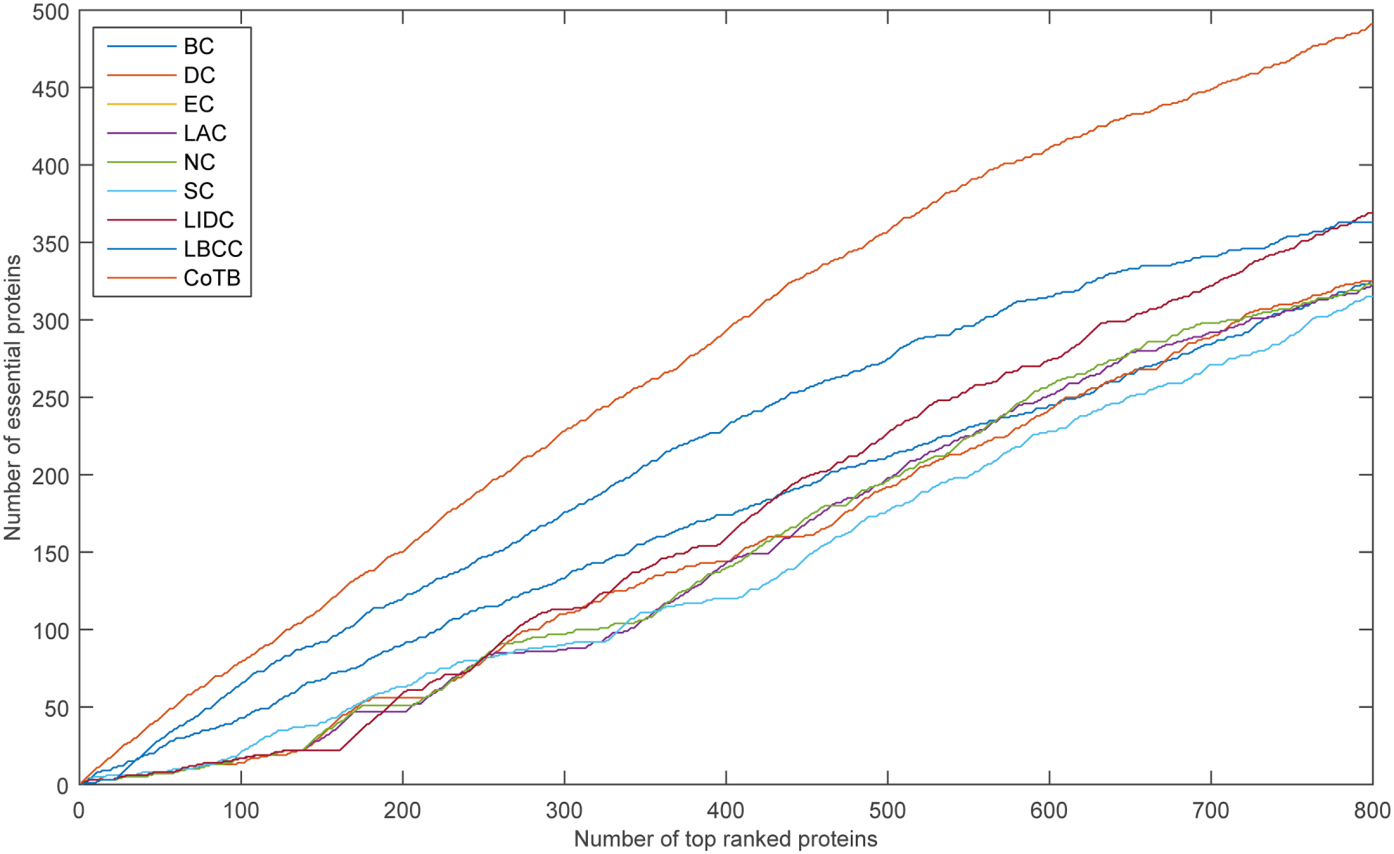
Jackknife curves of CoTB and the other methods for the YMBD network.

**Fig 12:**
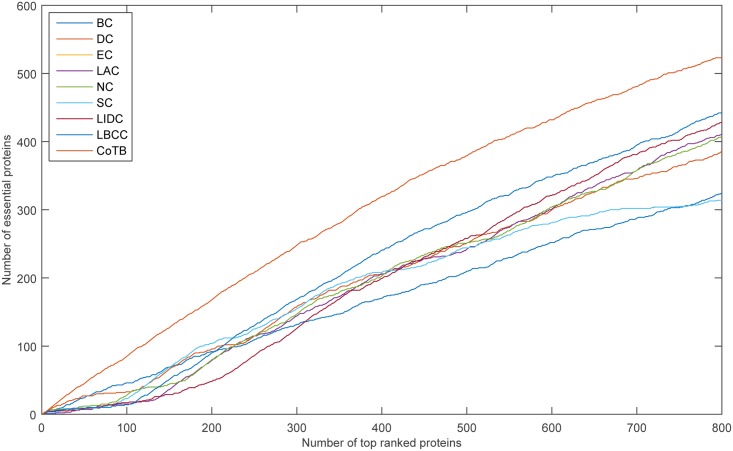
Jackknife curves of CoTB and the other methods for the YHQ network.

To further investigate the performance of CoTB, we used the jackknife methodology to assess the generality of our method. The x-axis represents the number of proteins ranked in descending order according to the values computed by the corresponding methods, and the y-axis represents the cumulative count of true essential proteins. The area under the curve is always used to measure the generality of a method. As shown in Figs 9–12, CoTB clearly performs better than BC, DC, EC, LAC, NC, SC, LIDC and LBCC on the four datasets, and it also performs better than SON on the YDIP dataset.

### Differences between CoTB and the other nine existing methods

To further analyse the differences between CoTB and the other nine existing methods, we compared the performances of the methods in predicting the top 100 proteins ranked by the corresponding methods on the YDIP dataset (see the supplementary S3 Excel). The overlapping rates of proteins predicted by CoTB and the other nine methods are presented in Table 3. Compared to the traditional methods that use topological features, such as BC, DC, EC, LAC, NC and SC, the overlapping rates are less than 19 percent. Compared to LIDC, LBCC and SON, the overlapping rates are 46, 37 and 25 percent, respectively. It is clear that CoTB significantly differs from the traditional methods, and it takes more biological knowledge into account, which helps locate essential proteins more accurately and stably.

**Table 3:** The overlapping rates of the proteins predicted by CoTB and the other nine methods for the YDIP network.

Method	BC	DC	EC	LAC	NC	SC	LIDC	LBCC	SON
Overlapping rate	3%	4%	3%	19%	13%	3%	46%	37%	25%

Subsequently, we analysed the top 100 proteins identified by LIDC, LBCC, SON and CoTB on the YDIP dataset. For LIDC and CoTB, 46 of the same proteins are identified by these methods, and for the remaining 54 proteins, CoTB identified 45 true essential proteins, whereas LIDC identified 36 true essential proteins. For LBCC and CoTB, 37 of the same proteins are identified by these methods, and for the remaining 63 proteins, CoTB identified 54 true essential proteins, whereas LBCC identified 39 true essential proteins. For SON and CoTB, 25 of the same proteins are identified by these methods, and for the remaining 75 proteins, CoTB identified 66 true essential proteins, whereas SON identified 58 true essential proteins.

Therefore, the comparative results demonstrate that CoTB is distinctly different from the other methods, and it can identify more true essential proteins.

Moreover, we also conducted experiments on each of the four datasets (YDIP, YMIPS, YMBD, and YHQ) through 10-fold cross-validation. The average AUC values are listed in Table 4. For the YDIP dataset, we have listed the AUC values of GEP and Acencio as mentioned in the paper [42]. CoTB obtains the best performance among GEP and Acencio. For the other datasets, we have listed the average AUC of CoTB. The results further demonstrate that CoTB is an effective method for identifying essential proteins.

**Table 4:** Comparison of CoTB with other methods.

Dataset	Methods	AUC
YDIP	**CoTB**	**0.788**
	GEP	0.773
	Acencio	0.778
YMIPS	CoTB	0.808
YMBD	CoTB	0.788
YHQ	CoTB	0.802

### Results on human PPI network

To further assess the performance of the CoTB method, we also conducted experiments on a human PPI network. The human PPI network data, denoted HDIP, were downloaded from the DIP database [30]. The protein complex set, denoted HCOM, was downloaded from CORUM [43]. The essential proteins were downloaded from DEG [32]. The dataset of orthologous proteins was downloaded from the InParanoid database [39] containing 71 reference organisms. Finally, the subcellular localization information was downloaded from the COMPARTMENTS database [40]. HDIP consists of 4647 interactions and 2914 proteins, including 1887 essential proteins, and HCOM contains 1283 protein complexes.

**Fig 13:**
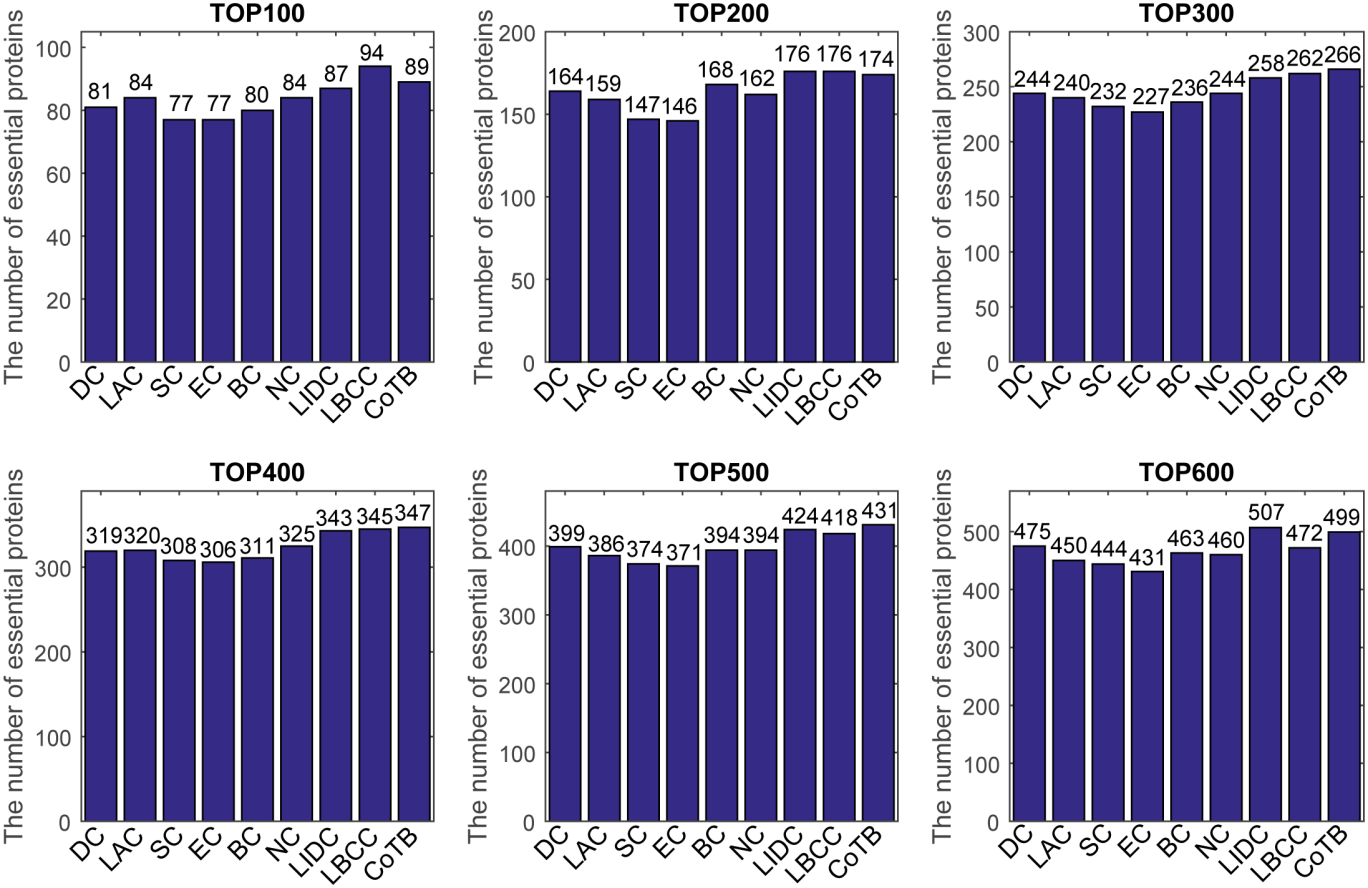
The number of essential proteins predicted by CoTB and the other eight methods at six levels for the HDIP network.

We used the four different YDIP, YMIPS, YMBD and YHQ datasets as the training set and the HDIP dataset as the testing set. First, we compared the performances of CoTB and the other eight methods at six levels from the top 100 to top 600. As shown in Fig 13, CoTB achieved the best results at the top 300-500 levels and exhibited performance similar to that of the methods attaining the best results at the top 100, 200 and 600 levels.

**Fig 14:**
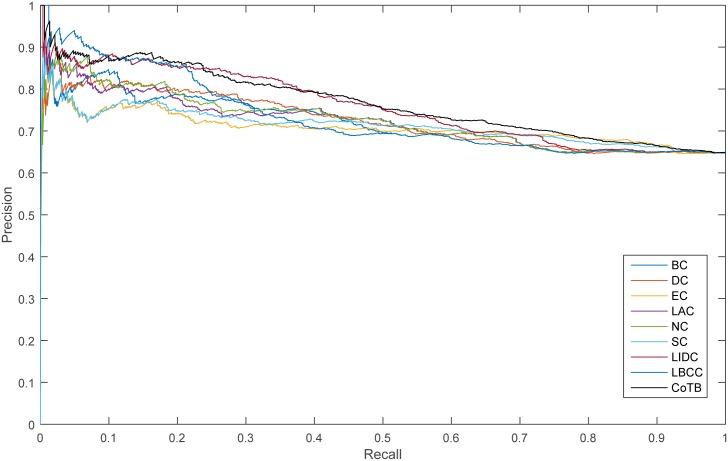
PR curves of CoTB and the other eight methods for the HDIP network.

**Fig 15:**
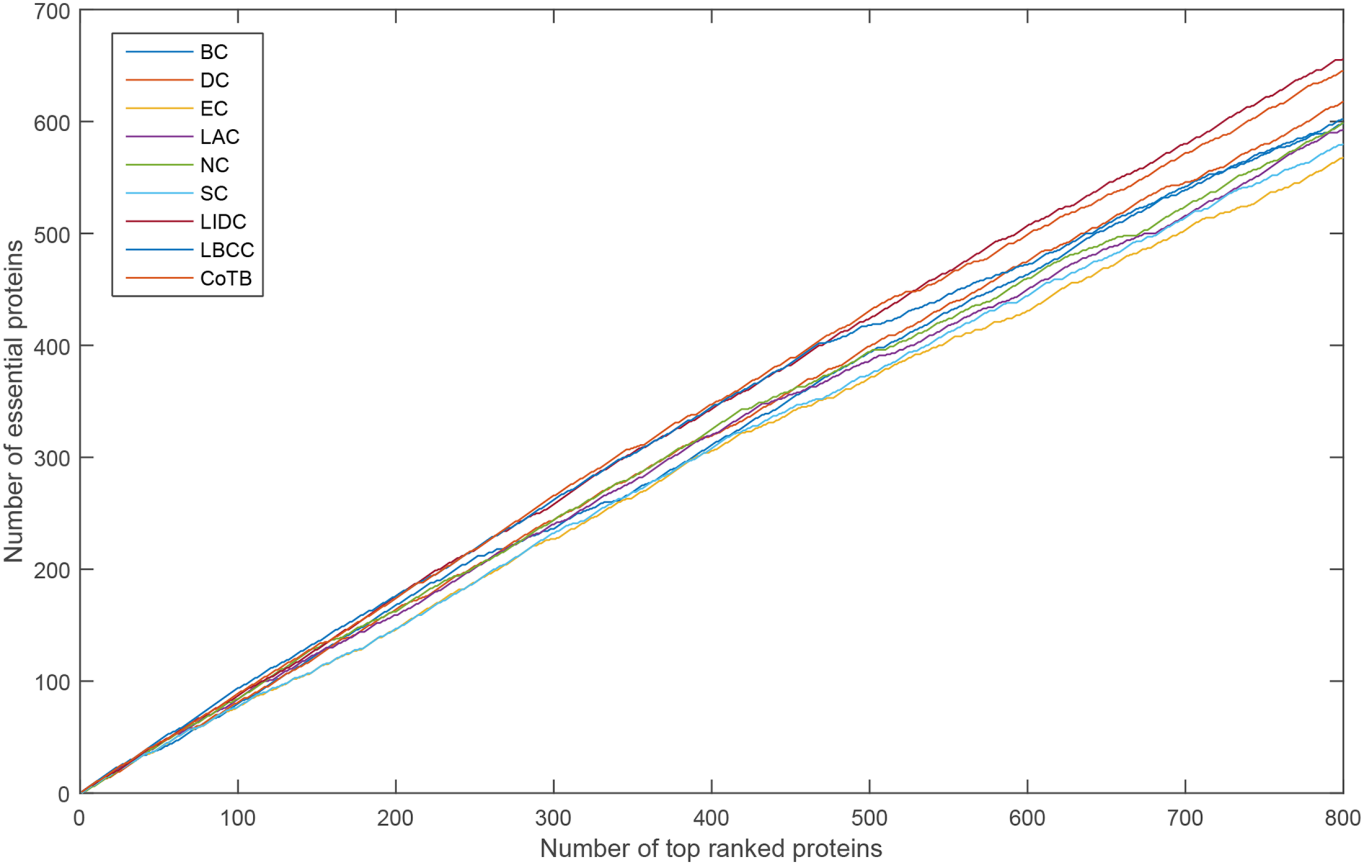
Jackknife curves of CoTB and the other eight methods for the HDIP network.

Then, we used six statistical measures, precision-recall curves and jackknife curves to evaluate the performance of the proposed CoTB method and the other eight methods. As shown in Table 5, the values of the six statistical measures for CoTB were slightly lower than for LIDC. From the precision-recall curves shown in Fig 14, CoTB obtained better performance between the recall levels of 0.11-0.22 and 0.38-0.82. From the jackknife curves shown in Fig 15, CoTB exhibited performance similar to that of LIDC and LBCC before the top 280 and achieved better performance between the top 280 to top 530. Hence, CoTB is also an effective method for discovering essential proteins for the human PPI network HDIP.

**Table 5:** Comparative analysis of CoTB and the other methods in terms of SN, SP, PPV, NPV, F-measure, and ACC on the HDIP dataset.

Dataset	Methods	SN	SP	PPV	NPV	F-measure	ACC
HDIP	DC	0.244	0.882	0.792	0.389	0.373	0.469
	LAC	0.232	0.860	0.753	0.379	0.355	0.453
	SC	0.230	0.856	0.746	0.377	0.352	0.451
	EC	0.223	0.843	0.723	0.371	0.341	0.442
	BC	0.240	0.873	0.777	0.385	0.366	0.463
	NC	0.235	0.866	0.763	0.381	0.360	0.457
	**LIDC**	**0.262**	**0.914**	**0.849**	**0.403**	**0.400**	**0.492**
	LBCC	0.245	0.884	0.796	0.389	0.375	0.470
	CoTB	0.257	0.906	0.833	0.399	0.393	0.486

## Conclusion

Identifying essential proteins is of great importance for understanding the molecular mechanisms of cellular life. Many computational methods combined with biological information have recently been proposed for this purpose. In 2016, Qin [17] proposed a method named LBCC based on the combination of topological features and protein complex information. This method improved the prediction accuracy to 74 percent on the YDIP dataset. Li [23] proposed a method named SON that uses a combination of topological features and biological information. This method improved the prediction accuracy to 81 percent on the YDIP dataset.

In this paper, we propose a new computational strategy named CoTB to predict essential proteins. First, we introduce several topological properties. Second, we propose new measures of orthologous score (DOS) and subcellular localization score (SLS), as well as a new computational strategy that combines Den_1_, Den_2_, BC, IDC, LC, DOS and SLS and uses a random forest prediction model to obtain a probability score for the proteins being essential. Finally, we apply CoTB on four networks of *Saccharomyces* *cerevisiae* and perform comprehensive comparisons of CoTB with nine other previously proposed methods. The results at six levels from the top 100 to top 600 demonstrate that our new method, CoTB, is more accurate than the other methods. Compared to the recently developed method SON, CoTB improves the prediction precisions by more than 9, 10, 8, 8, 7, and 8 percent at six levels on the YDIP dataset. Compared to the recently developed method LBCC, CoTB increases the prediction precisions by more than 4, 6, 11, 18, 14, and 16 percent at six levels on the other three datasets. In particular, CoTB improves the prediction precisions to 89, 78, 79, and 85 percent at the top 100 level on the YDIP, YMIPS, YMBD, and YHQ datasets, respectively. From the analysis of the six statistical measures, PR curves and jackknife curves, we find that CoTB is significantly superior to the other methods. Moreover, we also applied CoTB to a human PPI network, HDIP. The experimental results show that CoTB is also an effective method for predicting essential proteins for the HDIP network. There are two reasons leading to the outstanding performance of CoTB: the first reason is that it combines topological properties (both local and global properties) unlike SON and biological information (both subcellular localization and orthologous proteins) unlike LBCC. The second reason is that the machine learning method, random forest, plays an important role in the process of using these attributes to predict essential proteins. In conclusion, CoTB is a more effective, stable, and accurate method for predicting essential proteins.

## Supporting information

S1 Text Protein interaction data in YDIP. (TXT)

S2 Text Protein interaction data in YMIPS. (TXT)

S3 Text Protein interaction data in YMBD. (TXT)

S4 Text Protein interaction data in YHQ. (TXT)

S1 Excel Essential protein and nonessential protein data. (XLS)

S2 Excel Protein complex data. (XLSX)

S3 Excel The top 100 proteins obtained by the corresponding methods. (XLSX)
